# A relationship exists between replicative senescence and cardiovascular health

**DOI:** 10.1186/2046-2395-2-3

**Published:** 2013-02-04

**Authors:** Maria E Karavassilis, Richard Faragher

**Affiliations:** 1 School of Medicine, The Commonwealth Building, The Hammersmith Hospital, Imperial College London, Du Cane Road, W12 0NN, London, UK; 2 School of Pharmacy and Biomolecular Sciences, University of Brighton, Huxley Building, Lewes Road, BN2 4GJ, Brighton, UK

**Keywords:** Replicative senescence, Vascular endothelial cells, Vascular smooth muscle cells, Ageing, Atherosclerosis, Hypertension

## Abstract

A growing body of evidence demonstrates that the accumulation of senescent cells is a plausible ageing mechanism. It has been proposed that the senescence of vascular cells plays a causal role in the development of cardiovascular pathologies. A key prediction arising from this hypothesis is that cultures of cells derived from donors with cardiovascular disease will show reduced *in vitro* replicative capacities compared to those derived from disease-free controls. Accordingly, we carried out a formal review of the relationship among donor age, cardiovascular health status and maximum population doubling level attained *in vitro* by cultures of vascular smooth muscle and endothelial cells. Data were available to us on a total of 202 independent cell cultures. An inverse relationship was found to exist between replicative capacity and donor age in both endothelial and vascular smooth muscle cells. Cultures derived from donors with cardiovascular disease showed a lower overall replicative potential than age-matched healthy controls. In general the replicative potential at the start of the lifespan was found to be higher in those individuals without disease than those with disease and the difference in average cumulative population doublings (CPDs) in age-matched individuals in the two groups remained roughly constant throughout the lifetime. These results are consistent with the model in which the inherited replicative capacity of vascular cells is a stronger determinant of the onset of cardiovascular disease later in life, than wear-and-tear throughout the life course.

## Review

### Introduction

#### The purpose of this article

A detailed understanding of the mechanisms underlying cardiovascular disease has important implications for the reduction of mortality and morbidity in the aged population. For largely historical reasons, the majority of studies of replicative senescence have focused on human fibroblasts, as it was in these cell types that the phenomenon of cellular senescence was first reported. Unfortunately, this has produced a distortion of the evidence base, which renders a detailed understanding on the relationship replicative senescence and health status problematic. This is because very few age-associated pathologies are directly attributable to the dermal layer of the skin (or other fibroblastoid tissue layers, such as the corneal stroma).

However, a number of cell types exist in which a direct link between the senescent phenotype and age-associated disease is potentially easier to interrogate. Endothelial cell (EC) and vascular smooth muscle cell (VSMC) senescence in particular has been linked to the development of cardiovascular disease, specifically atherosclerosis [1-3]. The senescent phenotype has been proposed to result in an impaired ability to replace damaged or lost cells or to produce altered tissue microenvironments within the vessel [4]. Accordingly we reviewed the available data on proliferative capacity, donor age and cardiovascular disease status with regard to these cell types, in order to produce a meta-dataset spanning a range of age groups and cardiovascular disease states, allowing provisional conclusions to be drawn.

#### The cell hypothesis of ageing

The hypothesis that the progressive accumulation of senescent cells with tissue turnover during life plays a causal role in ageing was first proposed more than 40 years ago and has gained increasing credibility in recent years [5]. Classic studies in this area include that of Martin *et al.*[6], who reported a reduction of approximately two population doublings per decade in fibroblasts derived from individuals aged from new-born to a hundred years, and that of Schneider and Mitsui [7], who demonstrated a reduced average fibroblast migration rate, lower replication rate and saturation density at confluence, in fibroblast cultures derived from older individuals compared to their younger counterparts.

However, several studies using fibroblast cultures have demonstrated no statistically significant decline in replicative potential with age [8,9]. One explanation for lack of correlation is the exclusive selection of healthy donors. Because senescent cells are highly likely to be causal agents of age-related disease [5], it follows that these may be absent in a study population comprising only healthy donors.

Maier and Westendorp (2008) [10] proposed that the relationship between senescence and ageing is attributable to publication bias, and is absent in studies comprising large sample populations. However, this work contains questionable methodological aspects, the major constraint being the use of data derived from the fibroblast literature. Skin samples are simply not directly reflective of the pathological mechanism underlying Alzheimer’s disease.

There are literature reports where the proliferative capacity of fibroblasts from centenarians is not significantly reduced compared to those from younger donors [11]. This may reflect the fact that fibroblast replicative capacity in culture is not representative of the key organ systems *in vivo*.

The notion that senescence is a causal process driving at least some aspects of ageing is undeniable, as senescent cells have been found to be present and accumulate in tissue *in vivo* as a function of organismal age [12,13]. Among the best evidence of this is provided by a study where the number of mitotic cells in the proliferative region of murine lens epithelium was seen to decline with the age of the organism, with a concurrent increase in senescent cells [14].

The presence of senescence-associated markers at sites of pathology supports the relationship between replicative senescence and age-related disease. Using senescence-associated beta-galactosidase, senescent endothelial cells have been found to accumulate after repeated balloon endothelial denudation of the rabbit carotid artery [15]. It has also been demonstrated that telomeres in the endothelium shorten with age and this is more pronounced in atherosclerosis-prone areas [14-16]. Moreover, cultures of vascular smooth muscle cells derived from plaques have been reported to show a reduced proliferative capacity [17].

The senescent phenotype of vascular cells is not inconsistent with the potential to cause cardiovascular disease. Senescent endothelial cells *in vitro* have been found to over-express proteins characteristic of the pro-inflammatory and pro-thrombotic phenotype of the endothelium in human atherosclerosis, including IL-1alpha, ICAM-1 and PAI-1 [18-20]. Burton *et al.* (2009) demonstrated that key genes known to be up-regulated in atherosclerotic plaques are also highly up-regulated in senescent VSMCs. Most importantly, it was found that senescent VSMCs adopt a phenotype which contributes to the pathogenesis of vascular calcification, characterized by the expression of genes associated with vascular calcification, namely matrix Gla protein, bone morphogenetic protein-2, osteoprotegerin, osteopontin and decorin [21].

#### Hypothesis

**Figure 1:**
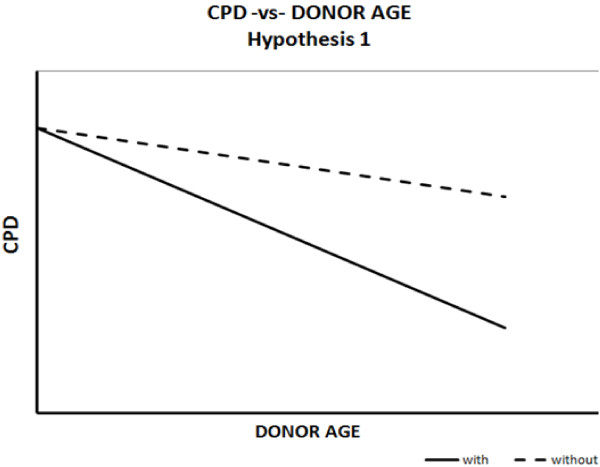
**Hypothesis 1: diseased and healthy individuals start with similar replicative capacities.** Replicative capacity is exhausted at a faster rate in diseased individuals.

The relationship between senescence and disease state throughout the lifetime can be modeled in three ways. In the first hypothesis, all individuals are expected to show similar inherited replicative capacities at the start of their lifespan. In subjects without cardiovascular disease, senescence is assumed to be the consequence of lifelong reparative cell divisions with advancing age [22-24]. In subjects with cardiovascular disease, senescence may be thought to represent an acceleration of the biological ageing process, triggered by exposure to mitotic stress, oxidative stress or DNA damage, independent of chronological age [25-27]. Those more prone to developing disease are, therefore, expected to exhaust their replicative potential at an accelerated rate compared to healthy individuals. The expected graphical relationship shows the trend lines of diseased and healthy individuals originating on the same point on the Y-axis, from which they diverge, with the line representing diseased individuals showing a steeper slope (Figure 1).

**Figure 2:**
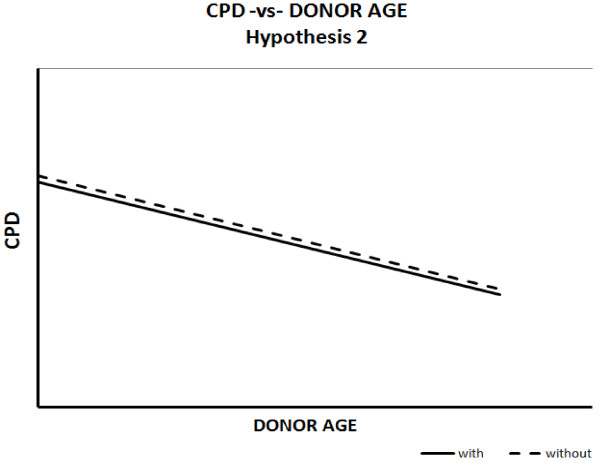
Hypothesis 2: diseased and healthy individuals coincide in terms of replicative capacity throughout lifespan.

It may otherwise be the case that replicative capacity always declines consistently with chronological age, regardless of health status (Figure 2). In this case, the expected graphical relationship should show that both trend lines coincide throughout the organism’s lifespan.

**Figure 3:**
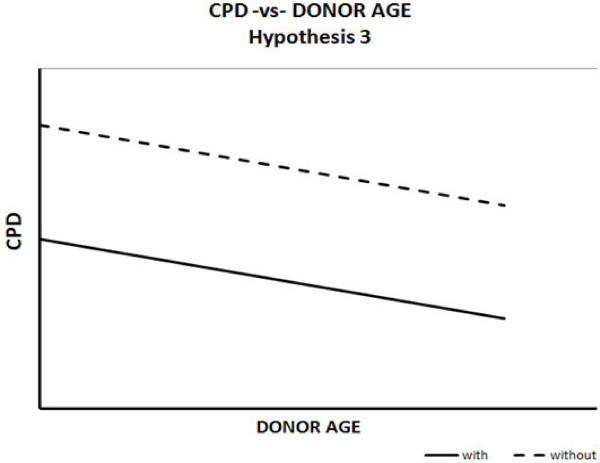
**Hypothesis 3: diseased and healthy individuals start off with different replicative capacities.** Replicative capacity is exhausted at similar rates.

An alternative hypothesis is that individuals differ in inherited replicative capacity at the start of their lifespan, but exhaust their replicative potential at a similar rate. Those more prone to developing disease are expected to start off with a lower replicative capacity (Figure 3). The expected graphical relationship should show the trend line corresponding to diseased individuals to start out at a lower point on the y-axis compared to non-diseased individuals, after which point the trend lines decline at similar rates, appearing to be parallel.

Individual differences in replicative capacity may be attributed to variations of inherited telomere lengths, as has been suggested in several studies. In one report, blood telomere lengths were found to range from 5.10 kb in young men from Italy to 18.64 kb in young men from Belgium, showing a greater than three-fold difference across populations [28]. In another study, individuals of African-ancestry in the USA tended to show 10% longer blood telomere lengths compared to those of European ancestry [29]. The large variation of telomere lengths observed in adults was suggested to relate to adaptive evolution, differences in early life experience or growth [30], exposure to stress throughout the lifespan [31] and, most pertinently in this case, varying paternal ages at reproduction. Kimura *et al.*[32] investigated the relationship between telomere length in sperm from young (30 years) and older (50+ years) donors with mean leukocyte telomere length in offspring (in adult ages); they demonstrated that a subset of sperm from older men had elongated telomeres, which corresponded to a positive relationship between paternal age at reproduction and offspring telomere length. The variation of telomere length in embryo-derived cells appears not to have been studied in detail.

Certainly, vascular cells with inherited short telomeres that are also exposed to considerable amounts of stress throughout the life course should show a much lower replicative potential compared to healthy individuals. The most likely hypothesis is, therefore, based on the notion that most disease states are multi-factorial in nature. When combining data from many individuals, it is expected that the relationship between replicative capacity and ageing in diseased individuals will show an overall lower cumulative population doubling (CPD) potential compared to healthy controls, as well as an accelerated rate of decrease of CPD.

### Materials and methods

#### Data mining

We mined the available peer-reviewed literature (principally using PubMed and relevant electronic journals directly). Combinations of search terms used included: ‘endothelium’, ‘vascular smooth muscle cells’, ‘senescence’, ‘cumulative population doublings’, ‘*in vitro*’, ‘passage’ and ‘donor age’. The search covered over 10,000 research articles. When available, the following information was obtained: age of donor, replicative capacity *in vitro*, vessel of origin, gender, incidence of cardiovascular-related disease and definition of replicative senescence. Articles were excluded if replicative capacity *in vitro* was recorded in such a way that CPD could not be accurately deduced. Ultimately, 12 research articles were used, published between 1978 and 2008.

#### Replicative capacity and health status

Studies were included in this review if donor age and replicative capacity of EC or VSMC were recorded. If not given, the CPD value was extrapolated from maximum passages achieved (given the cell culture split ratio) or approximated from scatter plots. If health status was not mentioned, it was assumed that the donors did not suffer any cardiovascular-related diseases.

#### Statistical analysis

To assess the strength of the correlation between *in vitro* replicative capacity and age of donor, regression analyses were carried out for each cell type, as well as sample populations with/without vascular–related diseases separately. For endothelial cells, graphs including all data points as well as graphs excluding human umbilical vein endothelial cells (HUVECs) are shown, as HUVEC samples show a very large scatter, which affected the trend lines. The mean decade CPD was plotted against donor decade age, to balance out any effects from possible confounding variables.

The mean rate of change of CPD with respect to age (change in CPD per year) was calculated for the graphs of: ‘all CPD values versus donor age’, ‘mean CPD versus donor age decade’ and for the graphs of ‘CPD versus donor age’ in those with or without vascular-related diseases shown separately.

To assess the contribution of confounding factors, scatter plots were drawn to visualize clustering of data points, per vessel type and per gender.

T-tests were performed to assess whether the difference in mean CPD for adjacent decades as well as decades at either extreme, could be attributed to chance. A T-test was also carried out comparing CPD of male versus female donors for each cell type.

### Results

#### Replicative capacity in vitro versus age of donor

The linear regression line shows a clear inverse relationship between replicative capacity *in vitro* and donor age, indicating a negative correlation for VSMC and EC. For both cell types, it is evident from the graphs that in the 50+ age group there are a larger proportion of points with low CPD values, that is, below 10 CPD in VSMCs and below 20 CPD in ECs.

**Figure 4:**
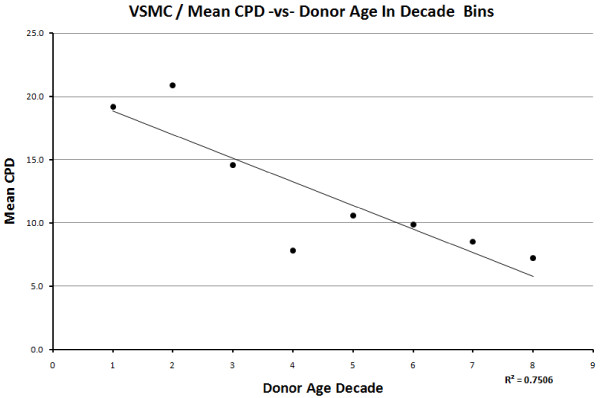
Relationship between the decade mean CPDs and donor age decade in vascular smooth muscle cells.

**Figure 5:**
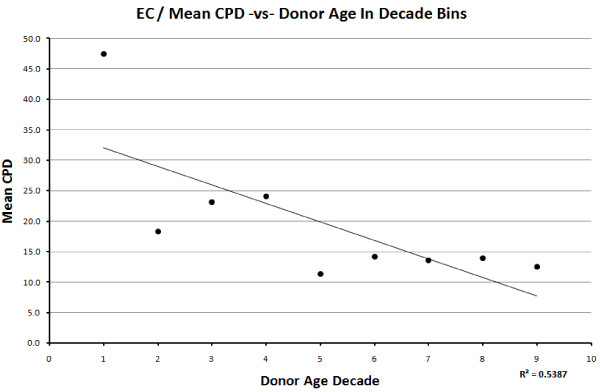
Relationship between the decade mean CPDs and donor age decade in endothelial cells.

When the decade mean CPD is plotted against donor age, a negative correlation is seen for both cell types, with R = −0.866 for VSMC (Figure 4), R = −0.733 for EC (Figure 5).

T-tests were carried out between datasets for adjacent decades. The *P*-values for the comparisons between adjacent decades in both cell types (with the exception of decades 4 and 5 in EC), indicate that one cannot say with certainty that the observed difference for adjacent decades cannot be attributed to chance (Table 1). The *P*-values comparing the first and eighth decades indicate that the differences are highly significant. The same conclusion was drawn when comparing the first and last two and three decades in VSMC and EC respectively. Thus, based on the available dataset, although it is not strongly evidenced that small age differences show a decrease in CPDs, a large age difference is clearly associated with a substantial decrease in CPD.

**Table 1:** **Table showing cell type, decades being compared, *P*-values and T-test interpretation**

**Cell type**	**Decades being compared**	***P*-value**	**T-test interpretation**
VSMC	1, 2	0.734	No Sig. Diff.
VSMC	2, 3	0.386	No Sig. Diff.
VSMC	3, 4	0.393	No Sig. Diff.
VSMC	4, 5	0.764	No Sig. Diff.
VSMC	5, 6	0.856	No Sig. Diff.
VSMC	6, 7	0.597	No Sig. Diff.
VSMC	7, 8	0.638	No Sig. Diff.
VSMC	1, 8	3.47E-04	Sig. Diff.
VSMC	1+2, 7+8	1.43E-05	Sig. Diff.
EC (all)	1, 2	0.0205	Sig. Diff.
EC (all)	2, 3	0.715	No Sig. Diff.
EC (all)	3, 4	0.947	No Sig. Diff.
EC (all)	4, 5	0.0235	Sig. Diff.
EC (all)	5, 6	0.444	No Sig. Diff.
EC (all)	6, 7	0.838	No Sig. Diff.
EC (all)	7, 8	0.887	No Sig. Diff.
EC (all)	8, 9	0.635	No Sig. Diff.
EC (all)	1, 8	4.04E-10	Sig. Diff.
EC (all)	1+2+3, 7+8+9	5.46E-12	Sig. Diff.
EC (excluding HUVECs)	2+3, 7+8+9	1.98E-03	Sig. Diff.

The mean rate of change in CPD with respect to age was also evaluated by calculating the slope of the linear regression line (Table 2). Table 3 shows the mean rate of change in CPD with respect to age, per publication. One can see that VSMC shows a lower rate of decrease per year (−0.19) compared to EC (−0.44) and similar rate of decrease when excluding HUVECs (−0.15). Looking at the mean decade CPD with respect to age decade, both cell types gave the same value (−0.19) of rate of change.

**Table 2:** Table showing cell type, variables and change in cumulative population doublings per year

**Cell type**	**Variables**	**Change in CPD per year**
EC	CPD values -vs-donor age (all)	−0.44
EC	CPD values-vs-donor age (excluding HUVECs)	−0.15
EC	Mean CPD values-vs-donor age decade (all)	−0.19
EC	Mean CPD values-vs-donor age decade (excluding HUVECs)	−0.19
VSMC	CPD values -vs-donor age	−0.19
VSMC	Mean CPD values-vs-donor age decade	−0.19
EC	CPD -vs- donor age in donors with cardiovascular-related diseases (all)	−0.14
EC	CPD -vs- donor age in donors without cardiovascular-related diseases (all)	−0.47
EC	CPD -vs- donor age in donors without cardiovascular-related diseases (excluding HUVECs)	−0.16
VSMC	CPD -vs- donor age in donors with cardiovascular-related diseases	−0.17
VSMC	CPD -vs- donor age in donors without cardiovascular-related diseases	−0.2

**Table 3:** Table showing cell type, publication, change in CPD per year, and number of samples

**Cell type**	**Variables**	**Change in CPD per year**	**No. of samples**
VSMC	Eskin (1981) [47]	−0.029	64
VSMC	Fukai (1994) [42]	−0.168	12
VSMC	Kan (1987) [48]	−0.176	4
VSMC	Bierman (1978) [49]	−0.03	17
EC	Hoshi (1986) [50]	−0.206	8
EC	Glassberg (1982) [51]	0	4
EC	Johnson (1992) [52]	−0.216	11
EC	Maciag (1981) [53]	NA	4
EC	Nobuhiko (1988) [54]	NA	2
EC	Vogel (2008) [37]	NA	2
EC	Watkins (1993) [41]	+0.45	2
EC	Vogel (2007) [55]	+0.119	61
**VSMC**	**TOTAL**	**−0.19**	**107**
**EC**	**TOTAL**	**−0.44**	**113**

#### Replicative capacity in vitro versus age of donor with respect to cardiovascular-related diseases

**Figure 6:**
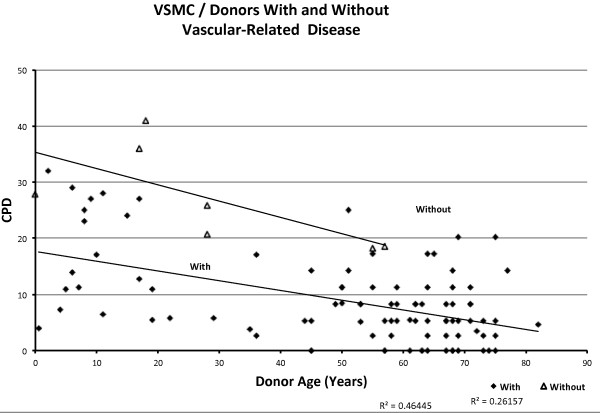
Relationship between CPDs and donor age in VSMCs, showing donors with and without cardiovascular-related diseases.

**Figure 7:**
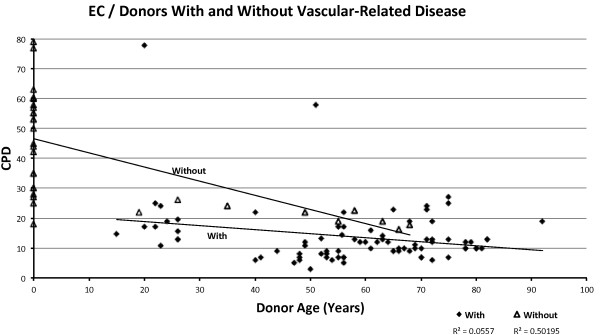
Relationship between CPDs and donor age in ECs, showing donors with and without cardiovascular-related diseases.

**Figure 8:**
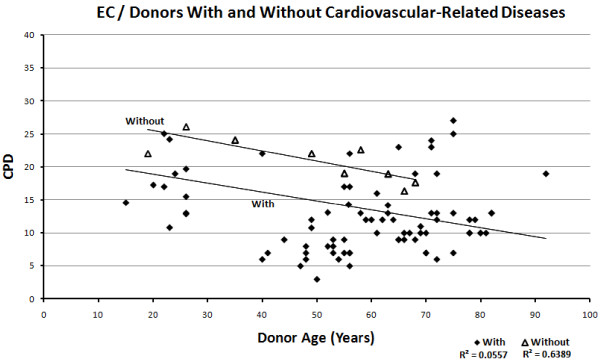
Relationship between CPDs and donor age in ECs, showing donors with and without cardiovascular-related diseases, excluding HUVEC data points.

The data points from donors with cardiovascular-related diseases show a negative correlation with age, with R = −0.511 in VSMCs (Figure 6) and R = −0.24 in EC (Figure 7). The data points from donors without cardiovascular related disease also show a negative but slightly weaker correlation with age R = −0.411 in VSMC (Figure 6) and much stronger correlation with age R = −0.708 in EC (Figure 7) and −0.799 when excluding HUVEC (Figure 8). It is useful to exclude HUVEC data points as they show a very wide scatter. Comparing the trend lines visually, it is clear that on average, CPDs are higher in those without cardiovascular-related disease compared to those with the disease for both cell types (when excluding HUVEC).

The T-tests between the sample populations with or without cardiovascular-related diseases show extremely small *P*-values of 3.89E-11 for VSMC and 1.21E-14 for EC and 1.11E-2 for EC, excluding HUVEC, indicating with a high degree of confidence that the observed difference between the means of the two data sets is not attributed to chance.

The change in CPD with respect to age in those without cardiovascular-related disease (Table 2) shows a slightly lower rate of decrease per year compared to those without cardiovascular-related disease, in both cell types, that is, -0.14 in ECs from donors with cardiovascular-related diseases, -0.47 in ECs without cardiovascular-related diseases, -0.16 in ECs without cardiovascular-related diseases, excluding HUVECs, -0.17 in VSMCs from donors with cardiovascular-related diseases, -0.2 from donors without cardiovascular-related diseases.

**Figure 9:**
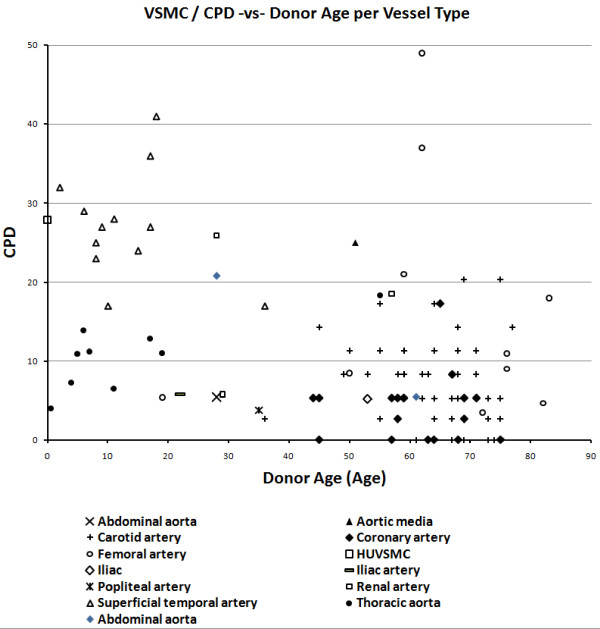
Relationship between cumulative population doublings and donor age in vascular smooth muscle cells, per vessel type.

**Figure 10:**
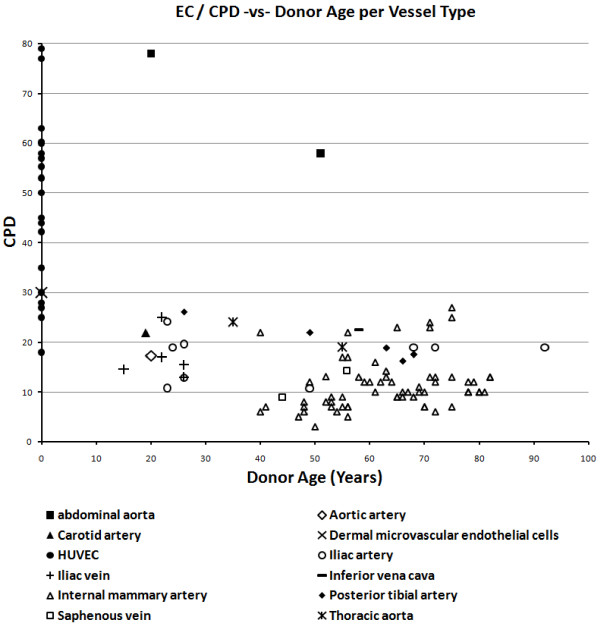
Relationship between cumulative population doublings and donor age in endothelial cells, per vessel type.

**Figure 11:**
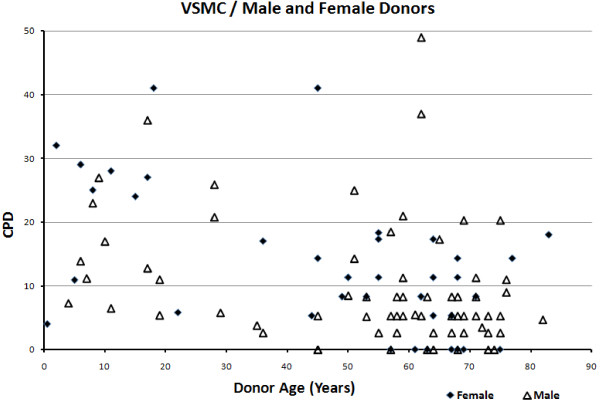
Relationship between cumulative population doublings and donor age in vascular smooth muscle cells, per gender.

**Figure 12:**
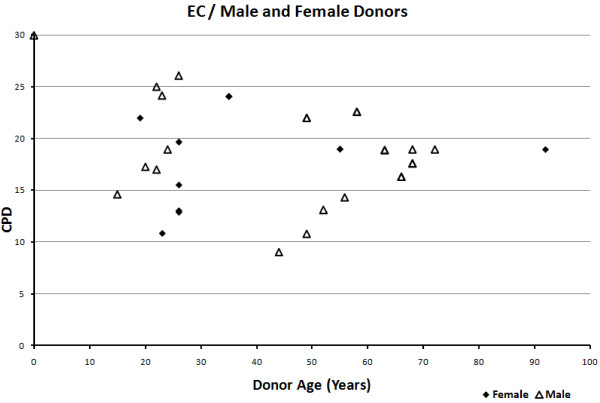
Relationship between cumulative population doublings and donor age in endothelial cells, per gender.

Scatter plots were drawn to assess the effects of vessel type and gender on EC and VSMC replicative capacity (Figures 9, 10, 11, 12). Overall, the data-points are seen to be equally spread in both cell types, regardless of gender or vessel type.

### Discussion

#### Replicative capacity in vitro versus age of donor

For both cell types there are fewer data points for the younger ages of 0 to 30 years, which is to be expected, as there is less availability for vessel biopsies (with the exception of HUVECs). Comparing the graph of the VSMCs with those of the ECs, the observed trends and average CPD values per age are roughly similar between the two cell types.

The data presented here are consistent with previous findings, showing a definite decline in proliferative activity *in vitro* with donor age. If the mean decade CPD values are used (to balance out the effects of possible confounding factors), the change in CPDs with age of both cell types corresponds to the finding by Martin, *et al.*[6] of approximately two CPDs per decade in fibroblasts. The findings are also strongly consistent with Hayflick’s observations on fibroblasts (1965) [33]; ECs derived from human embryos underwent about 48 (mean average) CPDs *in vitro*, ranging from 18 to 79 CPDs, while ECs derived from adults underwent roughly 20 (mean average) CPDs, ranging from 3 to 30 CPDs (excluding the two extreme outliers). Hayflick and Martin also emphasized the lack of correlation in the proliferative ability of cells derived from individuals of intermediate ages, despite the clear difference in the proliferative ability of cells derived from very old (70+) or very young (0 to 10) donors. The same conclusions were drawn in this study, as demonstrated by the T-test calculations between adjacent and extreme ages. The exception to this is the difference in CPDs between decades 4 and 5 in ECs. The graph shows a very clear and sudden decrease in replicative capacity between the two decades and the *P*-value shows a significant difference between the two datasets. A reason for this may be that the decline in replicative capacity as a function of age may not occur at a consistent rate, possibly showing an abrupt decrease at certain points in the lifetime. The relationship between *in vitro* replicative capacity and organismal ageing should, therefore, not be interpreted to be linear or very tight [34].

#### Replicative capacity in vitro versus age of donor, with respect to cardiovascular-related diseases

Many studies on vascular smooth muscle cells and endothelial cells have reported a relationship between the altered cellular phenotype associated with senescence and the onset of cardiovascular disease [18-21,35,36]. In this review, the graphs show a vivid relationship between cardiovascular-related disease and replicative capacity in vascular cells, supported by the T-test calculations. It is clear from the graphs that individuals with cardiovascular-disease show a lower cellular proliferative potential than those without cardiovascular disease. This suggests that disease state is more strongly associated with the onset of senescence than donor age.

Looking at the replicative capacity for HUVECs alone (limited data for embryo-derived VSMCs), the extremely wide scatter ranging from 18 to 79 CPDs already implies a variation in terms of inherited proliferative capacity, possibly attributed to differences in telomere length. Inherited telomere length is, in turn, thought to be affected by paternal age at reproduction [37]. If replicative capacity can be considered a heritable trait, it follows that neonates showing inherent low cellular proliferative capacity will be more prone to disease later in life. Since neonates cannot be distinguished on the basis of liability to disease (and because none are yet diagnosed with disease), including these in the graphical relation between disease and replicative capacity distorts the trend; classing all HUVECs as without disease is misleading, as these will show the trait assumed to underlie disease predisposition (low replicative capacity), as do the adult donors with disease.

When HUVECs are excluded, both ECs and VSMCs show that diseased individuals start off with a lower replicative capacity than non-diseased individuals. The difference in average CPDs in age-matched individuals in the two groups is roughly constant throughout the lifetime, that is, an average difference of 7 CPDs in ECs and 20 CPDs in VSMCs. This essentially supports the hypothesis suggesting that individuals differ in terms of inherited cellular replicative capacity from the start of their lifespan, but exhaust this proliferative potential at similar rates. These conclusions are in line with the assumption that inter-individual inherited telomere length varies widely. Substantial variations of telomere lengths have been reported in some studies [29,30,32]; however, the focus has been on adult donors. In adults, telomere shortening may occur following reparative cell divisions, exposure to stress or early infection, as well as differences in inherited telomere lengths. This probably accounts for the scattered distribution of data points corresponding to adult donors observed in this analysis. It would be more interesting if variation in telomere length of cells derived from human embryos were investigated in association with paternal age and telomere length in sperm. As far as we know, population variations of telomere length in neonates (that is, HUVECs) and with respect to paternal characteristics, has not yet been studied. Further exploration to elucidate the extent of variation of inherited telomere length and the possible consequences for disease, as initially implied in this review, is necessary. Despite the obvious inclination of these data toward inherited proliferative activity being strongly associated with disease state, the effect of decline in replicative capacity throughout the lifetime (that is, exogenous stress) should not be ignored. Cells from individuals with inherited short telomeres that are also exposed to considerable amounts of stress throughout the life course should undoubtedly show an even greater decrease in replicative capacity compared to healthy individuals.

While there is evidence that telomere shortening controls senescence in endothelial cells [3,38], it is unclear whether vascular smooth muscle cells undergo telomere-dependent senescence. It has been demonstrated that in VSMCs telomerase reverse transcriptase (TERT) is regulated at the transcriptional level, it is unclear whether over-expression of telomerase can bypass senescence [3,39]. There are limitations in terms of experimental approaches in these studies: each of these experiments involved analysis of pooled colonies, which is unreflective, since replicative capacity of cells in culture reflects the expansive propagation of the longest surviving clone. A more informative approach should involve isolation of as many colonies of vector-infected cells as possible (if possible, approximately n = 15). If all the isolated clones are found to be immortalized, the cell type can be described with a high certainty to have undergone telomerase-induced escape from senescence.

#### Limitations of this study

A quantitative relationship between proliferative capacity and age cannot be made with absolute certainty from these data. Nevertheless, the correlations found are surprisingly good, given that the studies included span over 30 years, with possible differences in donor characteristics, cell culture techniques, growth media, explanation methods and selection of biopsy areas.

Growth media are also well-recognized variables affecting cell growth and proliferation. Varying concentrations of serum used in culture result in different growth effects, as demonstrated by Holley *et al.*[40], with the number of PDs achieved directly related to the original concentration of serum added. Studies varied in terms of serum type and concentration used; for example, EC were cultured with 30% human serum in the study by Watkins *et al.*[41] while 15% fetal bovine serum was used in the study by Fukai *et al.*[42].

Studies also varied in terms of their definition of senescence, ranging from: senescence-associated morphological changes, 50% positive for SA-beta-galactosidase staining, or less than one PD occurring within three weeks after subculture. Diverse interpretations of ceased proliferation may have led to inconsistencies of the recorded replicative capacity.

Vessel type is an additional variable known to affect cellular replicative ability; for example, telomere length has been reported to decrease more rapidly in arterial rather than venous endothelial cells [15]. It has even been shown that the segment of the vessel used affects telomere attrition; for example, the distal versus the proximal segment of the abdominal aorta showing accelerated telomere attrition [16]. This most likely reflects the hemodynamic stress factor [43-46].

The scatter plots showing the effect of vessel type and gender showed the data-points to be generally equally distributed. Any exceptions were most likely due to factors associated with disease, shear stress or age, as different studies varied in terms of sample characteristics.

It would be misleading to perform a Funnel test (to test for publication bias) or a Cochrane-Q test (to test for heterogeneity between studies included). Different researchers focused on specific age ranges or disease states; therefore, any asymmetry observed in a funnel plot will probably not be due to systematic error or publication bias, but be due to factors associated with disease, age or sheer stress.

It should also be considered that studies included in this analysis did not always specify the site from which the cells were explanted. Moreover, the removal of vascular tissue is much less likely to occur in healthy individuals, compared to fibroblasts (which are far easier to obtain), unless the donor had died in an accident. It is, therefore, uncertain that all of the donors classed as ‘without cardiovascular-related disease’ included in this study were entirely healthy. For example, it is possible that senescence in endothelial or vascular smooth muscle cells is also dependent on damage induced by other, unrelated pathological conditions in the individual.

Despite all of this, the plots still show a clear inverse relationship between donor age and proliferative capacity *in vitro*. These conclusions are supported by the T-test values and correlation coefficients.

## Conclusions

This survey of the available literature demonstrates that a clear inverse relationship exists between the replicative capacity of vascular endothelial and smooth muscle cells and donor age. Individuals free of cardiovascular-related diseases show a greater replicative capacity at given ages, than those with defined pathologies. The mean CPD for each diseased age group roughly corresponds to that of older, non-diseased subjects. This suggests that disease state rather than donor age is the primary variable correlating with senescence. Perhaps surprisingly, individuals with cardiovascular disease appear to start off with a lower cellular replicative capacity in early life, than non-diseased individuals. The difference in average CPDs in age-matched individuals in the two groups is roughly constant throughout the lifetime. This is consistent with the hypothesis that individuals differ in terms of inherited cellular replicative capacity, but exhaust their proliferative potential at similar rates.

## Abbreviations

CPD: Cumulative Population Doublings; DNA: Deoxyribonucleic acid; EC: Endothelial Cell; HUVEC: Human Umbilical Vein Endothelial Cells; IL: 1α-Interleukin-1 alpha; ICAM-1: Intracellular Adhesion Molecule 1; NA: Not Applicable; PAI-1: Plasminogen Activator Inhibitor-1; TERT: Telomerase Reverse Transcriptase; VSMC: Vascular Smooth Muscle Cell.

## Competing interests

The authors declare that they have no competing interests.

## Authors’ contributions

RF devised, planned and assigned this project to MK to satisfy the criteria of completing a BSc. MK collected and analyzed the data and drafted the manuscript under the supervision of RF. All authors read and approved the final manuscript.
